# Cerebrospinal fluid dynamics modulation by diet and cytokines in rats

**DOI:** 10.1186/s12987-020-0168-z

**Published:** 2020-02-10

**Authors:** Zerin Alimajstorovic, Ester Pascual-Baixauli, Cheryl A. Hawkes, Basil Sharrack, A. Jane Loughlin, Ignacio A. Romero, Jane E. Preston

**Affiliations:** 1 School of Life, Health and Chemical Sciences, Open University, Walton Hall, Milton Keynes, MK7 6AA UK; 2 Department of Neuroscience, Royal Hallamshire Hospital, Glossop Road, Sheffield, S10 2JF UK; 3 Institute of Pharmaceutical Science, King’s College London, 3rd Floor, Franklin-Wilkins Building, 150 Stamford Street, London, SE1 9NH UK

**Keywords:** Idiopathic intracranial hypertension, Ventriculo-cisternal perfusion, Variable rate infusion, Raised intracranial pressure, Cytokines

## Abstract

**Background:**

Idiopathic intracranial hypertension (IIH) is a neurological disorder characterised by raised cerebrospinal fluid (CSF) pressure in the absence of any intracranial pathology. IIH mainly affects women with obesity between the ages of 15 and 45. Two possible mechanisms that could explain the increased CSF pressure in IIH are excessive CSF production by the choroid plexus (CP) epithelium or impaired CSF drainage from the brain. However, the molecular mechanisms controlling these mechanisms in IIH remain to be determined.

**Methods:**

In vivo ventriculo-cisternal perfusion (VCP) and variable rate infusion (VRI) techniques were used to assess changes in rates of CSF secretion and resistance to CSF drainage in female and male Wistar rats fed either a control (C) or high-fat (HF) diet (under anaesthesia with 20 μl/100 g medetomidine, 50 μl/100 g ketamine i.p). In addition, CSF secretion and drainage were assessed in female rats following treatment with inflammatory mediators known to be elevated in the CSF of IIH patients: C–C motif chemokine ligand 2 (CCL2), interleukin (IL)-17 (IL-17), IL-6, IL-1β, tumour necrosis factor-α (TNF-α), as well as glucocorticoid hydrocortisone (HC).

**Results:**

Female rats fed the HF diet had greater CSF secretion compared to those on control diet (3.18 ± 0.12 μl/min HF, 1.49 ± 0.15 μl/min control). Increased CSF secretion was seen in both groups following HC treatment (by 132% in controls and 114% in HF) but only in control rats following TNF-α treatment (137% increase). The resistance to CSF drainage was not different between control and HF fed female rats (6.13 ± 0.44 mmH_2_O min/μl controls, and 7.09 ± 0.26 mmH_2_O min/μl HF). and when treated with CCL2, both groups displayed an increase in resistance to CSF drainage of 141% (controls) and 139% (HF) indicating lower levels of CSF drainage.

**Conclusions:**

Weight loss and therapies targeting HC, TNF-α and CCL2, whether separately or in combination, may be beneficial to modulate rates of CSF secretion and/or resistance to CSF drainage pathways, both factors likely contributing to the raised intracranial pressure (ICP) observed in female IIH patients with obesity.

## Introduction

Idiopathic intracranial hypertension (IIH) is a neurological disorder characterised by raised intracranial pressure (ICP) and papilloedema in the absence of any other intracranial pathology or secondary cause [1, 2]. IIH typically affects women with obesity between the ages of 15 and 45, causing disabling daily headaches and visual loss, which is severe and permanent in up to 25% of cases [3]. While the pathogenesis is not yet known, it is thought that raised ICP is caused by an impairment of cerebrospinal fluid (CSF) drainage, or an increased production of CSF. CSF biomarkers have been used to present an insight into the pathogenesis of IIH.

Various risk factors have been postulated as mediators of IIH. Only obesity and female sex have been linked with a higher probability of developing the disease so far, however, it is thought that IIH is a multifactorial condition [4]. IIH presents a challenge to countries experiencing an obesity epidemic and it is thought that the incidence of IIH will increase following the trend in increased rates of obesity that is observed globally [5].

Cerebrospinal fluid is a clear fluid that surrounds the brain and spinal cord and is produced by the epithelial cells of the choroid plexus (CP). The CPs are anatomical structures in the third, fourth and lateral ventricles within the brain and are formed of blood vessels lined by the choroidal epithelium [6, 7]. The capillary endothelium in the CP is fenestrated and surrounded by these epithelial cells, joined by tight junctions, all of which constitute the blood–CSF-barrier (BCSFB) and control CSF composition [8].

It is thought that the main mechanism of CSF secretion is mediated by the CP epithelial cells. In brief, fluid secretion by the CP epithelial cells involves the sodium–potassium ATPase pump (Na^+^–K^+^-ATPase), Na^+^–K^+^–2Cl^−^ cotransporter (NKCC1), chloride channel (CIC-2) and aquaporin-1 water channel (AQP-1) located on the apical membrane, driving the efflux of Na^+^, Cl^−^, K^+^, HCO_3_^−^ and H_2_O from the blood into the CSF [6]. Further transporters, the chloride–bicarbonate exchanger (AE2) and sodium–bicarbonate cotransporters (NCBn1, NCBE), located on the basolateral membrane drive the accumulation of Na^+^, Cl^−^ and HCO_3_^−^ into the cytoplasm of the CP epithelial cells, eventually resulting in CSF secretion [6].

Once secreted, the CSF flows from the lateral and third ventricles before exiting through the fourth ventricle and into the subarachnoid space (SAS) or spinal cord. There are several pathways for CSF drainage but it is thought that CSF is predominantly absorbed via the arachnoid villi into the dural venous sinuses [9] or the nasal/dural lymphatics [10, 11].

The total volume of CSF within adult humans is approximately 140 ml and the rate of secretion by each CP is 0.2 ml/min [12]. The pressure required for the circulation of CSF is maintained by a hydrostatic pressure gradient between the CP (where CSF is produced) and the arachnoid villi (where CSF is drained) [8]. The CP epithelial cells are indispensable for directed transport processes from blood into the CSF, for the removal of substances out of the brain, and for CSF production [13].

An underlying inflammatory pathology is present in IIH in terms of abnormal expression of inflammatory mediators. This is because IIH is strongly associated with obesity, a chronic low grade pro-inflammatory state [14].

Cytokines are small, non-structural proteins that are synthesized by most nucleated cells. Cytokines include interleukins (IL), interferons and colony stimulating factors. Cytokines and chemokines (a sub-group of cytokines that direct chemotaxis in responsive cells) are involved in regulating inflammatory responses through coordination of cell movement to sites of infection [1]. Chemokines facilitate the passage of leukocytes from the circulation and into the tissues [15]. Due to its links with obesity, IIH may be associated with increased expression of adipokines and cytokines. Inflammatory mediators tumour necrosis factor-α (TNF-α) [16, 17], IL-6 [18], IL-17 [17] and C–C motif chemokine ligand 2 (CCL2) [1] as well as the glucocorticoid cortisol (hydrocortisone) [19] were found to be increased in the CSF and/or serum of IIH patients. Leptin and IL-1β were also studied but showed no significant results in either CSF secretion rates or resistance to CSF drainage. Pro-inflammatory cytokines could serve as important diagnostic markers of molecular pathways that may serve as targets for therapeutic intervention [1] if they are found to cause elevated ICP and therefore be a cause of IIH.

In addition to obesity being a factor in the incidence of IIH, sex hormones may influence patients with IIH as it is found to occur mostly in females with obesity.

The overall aim of this study was to investigate the effects of weight gain mediated by a high fat diet, and the acute effects of inflammatory mediators on CSF dynamics in the rat. In order to do that, CSF secretion in both control (C) and high-fat (HF) diet fed male and female rat models, and resistance to CSF drainage in female rats, with and without cytokine treatment were studied. These are the pathways thought to be the main problem causing increased ICP in IIH patients. HF diet fed female rats had the highest rate of CSF secretion. When treated with hydrocortisone (HC) and TNF-α, female rats also showed increased CSF secretion; as well as decreased CSF drainage following CCL2 treatment.

## Methods

### Animals

All in vivo techniques were performed at the School of Life, Health and Chemical Sciences, The Open University Milton Keynes, UK in accordance with Home Office project licence (PPL Number: 70/8507). Male and female Wistar rats were ordered Envigo, UK, at 4 weeks of age and maintained on either a C pellet or HF diet until sacrifice. Rats were fed either a C rodent maintenance 1 diet (5.7% fat, 14.4% protein, 79.9% carbohydrate, Table 1) (SDS, Essex, UK) or a HF diet (45% fat, 20%, protein and 35% carbohydrate, Table 2) (SDS, Essex, UK). The rats were fed ad-libitum and the diets were administered as pellets for a period of 7 weeks. Control rats and those receiving the HF diet were weighed every week from the onset of diet, and rat weights ranged between 250 and 300 g at the onset of experiments.

**Table 1:** Ingredients contained within % (w/w) of SDS RM1 rodent maintenance C diet

Control diet	
Ingredient	g% (w/w)
Fat	2.3
Carbohydrates	75.2
Protein	14.4
Fibre	4.7
Minerals	3.3
Vitamins	0.1
Total	100

**Table 2:** Ingredients contained within % (w/w) of SDS 45% AFE HF diet

High-fat diet	
Ingredient	g% (w/w)
Fat	38.6
Carbohydrates	30.1
Protein	20.1
Fibre	4.9
Minerals	4.6
Vitamins	1.7
Total	100

### In vivo CSF secretion—the ventriculo-cisternal perfusion technique

The ventriculo-cisternal perfusion (VCP) technique [20] was used in anaesthetised rats to measure in vivo CSF secretion rates. Artificial CSF (aCSF: 122 mM NaCl, 3 mM KCl, 1 mM CaCl_2_, 1 mM MgCl_2_, 15 mM NaHCO_3_, 15 mM HEPES, 0.5 mM Na_2_HPO_4_, 17.5 mM glucose) with 0.5% w/v blue dextran, containing the treatment of interest (concentration shown in Table 3) is perfused through the lateral ventricles and samples are collected over time from the *Cisterna magna* and analysed by spectrophotometry.

**Table 3:** Concentrations of the treatments added to the aCSF for ventriculo-cisternal perfusion and variable rate infusion experiments

Treatment	Supplier	Dose (ng/ml)	Reference based on reported levels in CSF of IIH patients
Hydrocortisone	Sigma-Aldrich, Dorset, UK (H0135)	500	Sinclair et al. [19]
TNF-α	Sigma-Aldrich, Dorset, UK (H8916)	0.1	Hayakata et al. [16]
IL-6	Life Technologies, Paisley, UK (10398-H08H-5)	0.1	Reihani-Kermani et al. [18]
IL-17	Miltenyi Biotech Ltd, Woking, UK (130-093-959)	0.1	Li et al. [41]
CCL2	Cambridge Bioscience, Cambridge, UK (00220-0-100)	50	Dhungana et al. [1]
IL-1β	Miltenyi Biotech Ltd, Woking, UK (130-093-897)	0.1	Hayakata et al. [16]
Leptin	Sigma-Aldrich, Dorset, UK (L4146)	100	Dhungana et al. [1]

The experiment was carried out in 11 week old male and female Wistar rats, 250–300 g. The animals were first anaesthetised using an isofluorane (Merial Animals Health, Essex, UK), administered within an inhalation chamber for 5 min. A single intraperitoneal injection of ‘Domitor’ (medetonidine hydrochloride) at 20 μl/100 g weight and ‘Vetalar’ (ketamine) at 50 μl/100 g weight of animal (both supplied by the Home Office Named Veterinary Surgeon, Red Kite Veterinary Consultants Centaur Services, Castle Cary, UK) was then given.

The head was held in position using a stereotaxic frame and a midline cutaneous incision was made from forehead to neck to expose the top of the skull. The lateral ventricles were located 0.8 mm posterior to the bregma and 1.5 mm laterally either side for each lateral ventricle. A 0.65 mm hand-chuck drill bit bore holes in the skull for insertion of metal cannulae to a depth of 4 mm. The cannulae were attached to a water manometer; a fall in pressure as the cannulae was inserted confirmed correct positioning within the ventricle and a pressure transducer was then connected to a side arm of the cannula to monitor infusion pressure.

A 1 mm diameter needle was inserted into the cisterna magna for collection of perfusion outflow. Entry into the cisterna magna was obtained by locating the base of the occipital bone, found at the back of the rat skull, before piercing the arachnoid membrane, below the bone, and inserting the needle into the SAS of the cisterna magna. Correct positioning of the needle was evident following immediate visualisation of aCSF (containing blue dextran) perfusion through the needle and into the 1 mm bore tubing.

Two 10 ml plastic syringes (14.5 mm diameter) were filled with aCSF containing the treatment/cytokine of interest (Table 3). Both lateral ventricles of the brain were perfused using a Harvard slow-drive syringe pump (Harvard Apparatus UK, Cambridge, UK, Cat No. 703007INT) for a total period of time of 90 min. Perfusion inflow rate of aCSF was 20 μl/min for each ventricle for the first 20 min and 10 μl/min for the remaining 70 min. The choice of perfusion rate was made to remove possible clots resulting from cannulae insertion and to rapidly flush out endogenous CSF, which was flushed out over the first 40 min. The need to reduce clot accumulation was an observation that was determined during the initial experiments CSF was sampled from the cisterna magna every 10 min to calculate CSF secretion rate based on Dextran dilution (ratio of Concentration out/Concentration in) measured in real time using Fluostar Optima, at 625 nm. Once steady-state Dextran dilution was achieved (by ≈ 60 min), secretion rates for subsequent samples were averaged until the end of the experiment (90 min) to calculate the CSF secretion rate for each animal.

The CSF secretion rate was calculated by the dilution of the blue dextran as shown in Eq. 1:

1 $${\text{CSF secretion rate }}\left({\upmu {\text{l/min}}} \right) = \frac{{{\text{C}}_{\text{in}} - {\text{C}}_{\text{out}} }}{{{\text{C}}_{\text{out}} }} \times {\text{Perfusion rate }}\left( {\upmu{\text{l/min}}} \right)$$

where C_in_ is the absorbance value of the initial aCSF (containing blue dextran) that was perfused into each of the lateral ventricles (concentration in) and C_out_ is the absorbance value of aCSF (containing blue dextran) that was perfused out of the cisterna magna (concentration out) for a particular perfusion period. The perfusion rate was the total of two syringes, i.e 2 × 10 μl/min.

### In vivo resistance to CSF drainage—variable rate infusion technique

This technique, used by Jones and colleagues measures the resistance to absorption of the CSF [21]. As with the VCP method, this technique was also carried out in female Wistar rats, 250–300 g. The animals were anaesthetised before being positioned into the stereotaxic frame as described previously.

One 10 ml plastic syringe was placed in the Harvard slow-drive syringe pump (Harvard Apparatus UK) (filled with aCSF with or without the treatment of interest). The concentrations of these treatments were the same as those used in VCP experiments, as described in Table 3.

During the variable rate infusion (VRI) technique, perfusion of the aCSF with the treatment of interest was undertaken through only one lateral ventricle of the brain in live anaesthetised rats. The other lateral ventricle was inserted with a cannula attached to a pressure transducer (Henley’s Medical Ltd, Herts, AL7 1AN UK), and pressure readings were taken at 10 min intervals at increasing perfusion rates. Infusion of aCSF at a known rate causes CSF pressure to rise to a plateau level. The resistance to absorption of the CSF was then calculated from the gradient of plateau pressure (recorded over four increasing rates), against the infusion rate.

### Statistical analysis

**Fig. 1:**
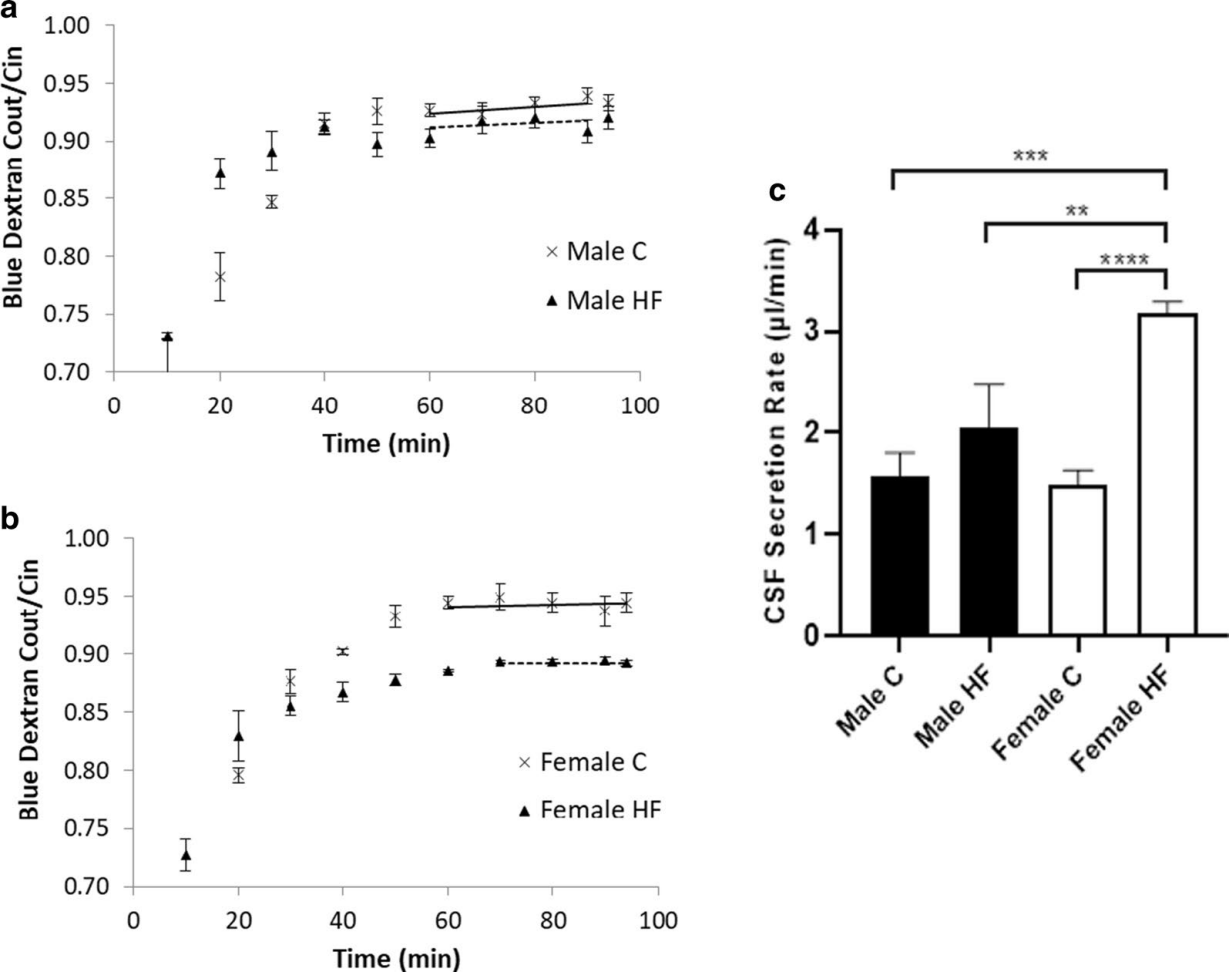
Control CSF secretion rates in male and female rats fed either a C or HF diet. Each VCP experiment was carried out by perfusing aCSF through both lateral ventricles of the rat brain. CSF secretion rates for **a** males and **b** females were calculated from the dilution of Blue Dextran (Blue Dextran C_out_/C_in_) after steady-state was reached shown by the lines (solid line control, dashed line high fat diet HF). **c** Samples from male C (n = 3), male HF (n = 4), female C (n = 3), female HF (n = 3) rats were averaged (±SD) and compared to one another. A two-way ANOVA was used to analyse the statistical significance. The significant results are shown following Sidak’s multiple comparison test a two-tailed equal variance t-test comparison each diet and sex variable. **P = ≤ 0.01, ***P = ≤ 0.001, ****P = ≤ 0.0001. *aCSF* artificial cerebrospinal fluid, *C* control diet, *HF* high-fat diet, *VCP* ventriculo-cisternal perfusion

**Fig. 2:**
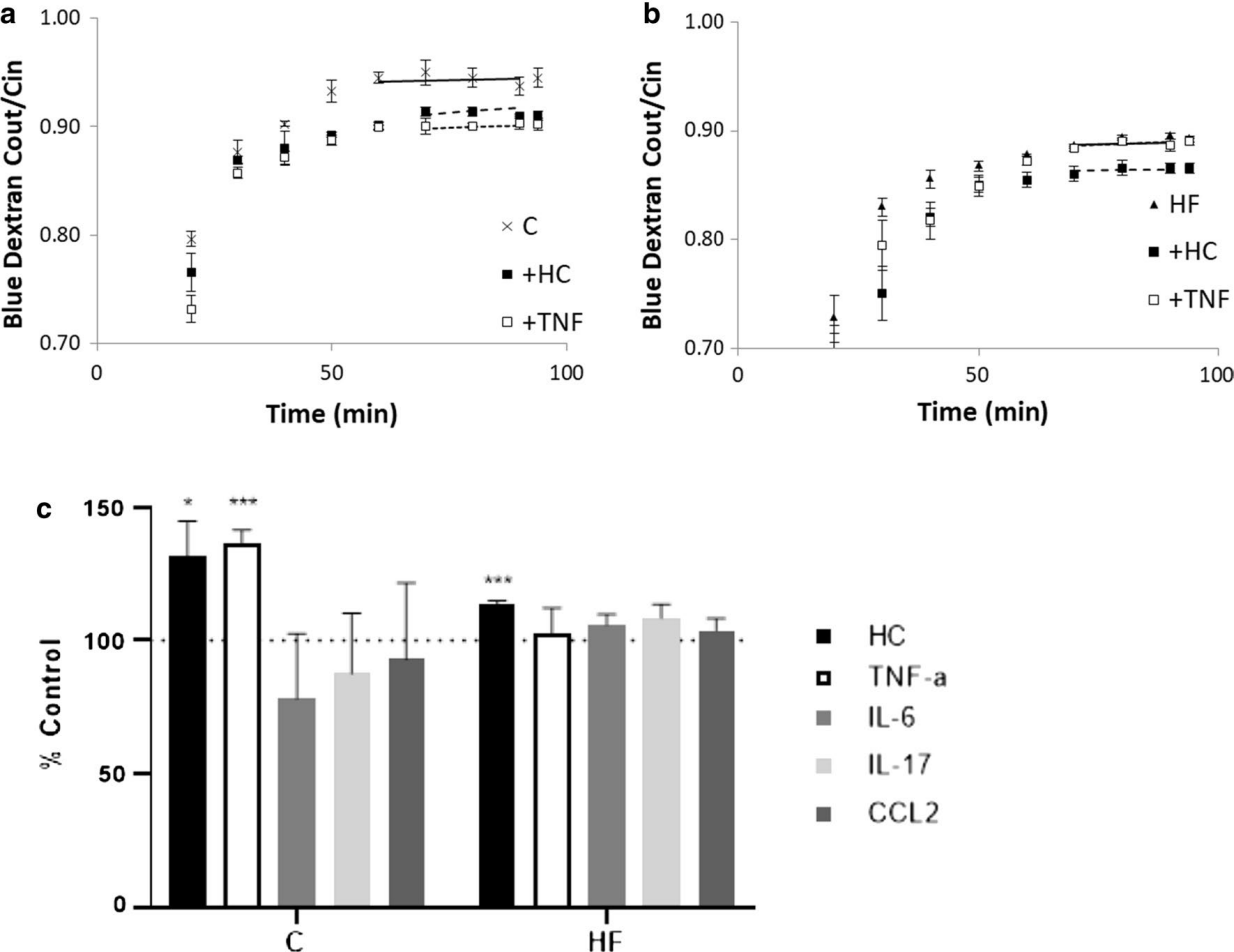
Effect of cytokine treatment on CSF secretion rate in female rats fed a C or HF diet. CSF secretion rates for **a** female controls and **b** female HF diet rats, were calculated from the dilution of Blue Dextran (Blue Dextran C_out_/C_in_) after steady-state was reached shown by the lines (solid lines no cytokines, dashed lines with cytokine). **c** CSF secretion rates were then calculated as a percentage change from each respective control (±SD) from female C (n = 3) and female HF (n = 3) rats for each respective cytokine treatment (n = 3). A two-way ANOVA was used to analyse the statistical significance. The significant results are shown following Sidak’s multiple comparison test a two-tailed equal variance t-test comparison each diet and sex variable. *P = ≤ 0.05, ***P=≤ 0.001. *aCSF* artificial cerebrospinal fluid, *C* control diet, *HF* high-fat diet, *VCP* ventriculo-cisternal perfusion, *HC* hydrocortisone, *TNF-α* tumour necrosis factor-α, *IL-6* interleukin-6, *IL-17* interleukin-17, *CCL2* C–C motif chemokine ligand-2

**Fig. 3:**
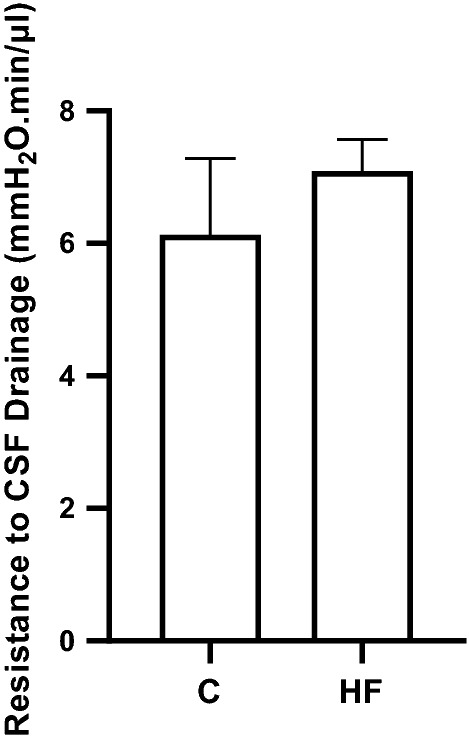
Control resistance to CSF drainage values in female rats fed a C or HF diet. Each VRI experiment was carried out by perfusing aCSF with each treatment through one lateral ventricle of the rat brain. Samples from each group were averaged (±SD); female C (n=3), female HF (n=3) and compared to one another. The graph shows the averaged resistance to CSF drainage readings (mmH_2_O min/μl). A one-way ANOVA was used to analyse the statistical significance. The significant results are shown following Sidak’s multiple comparison test and was performed against each diet. *aCSF* artificial cerebrospinal fluid, *C* control diet, *HF* high-fat diet, *VRI* variable rate infusion

**Fig. 4:**
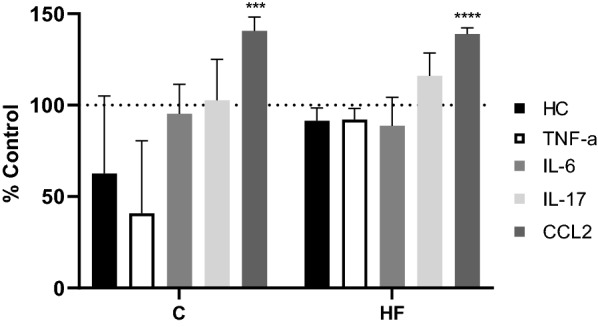
Resistance to CSF drainage values in female rats fed a C or HF diet with cytokine treatment. Each VRI experiment was carried out by perfusing aCSF with each treatment through one lateral ventricle of the rat brain. Values were calculated as a percentage change from each respective control from female C (n = 3) and female HF (n = 3) rats for each respective cytokine treatment (n = 3). The graph shows the averaged resistance to CSF drainage readings (mmH_2_O min/μl) as a percentage change from control. A two-way ANOVA was used to analyse the statistical significance. The significant results are shown following Sidak’s multiple comparison test and was performed for each diet and treatment variable. ***P = ≤ 0.001, ****P = ≤ 0.0001. *aCSF* artificial cerebrospinal fluid, *C* control diet, *HF* high-fat diet, *VRI* variable rate infusion, *HC* hydrocortisone, *TNF-α* tumour necrosis factor-α, *IL-6* interleukin-6, *IL-17* interleukin-17, *CCL2* C–C motif chemokine ligand-2

All data are presented as mean ± standard deviation of the mean and are the result of a number of independent experiments (n) with replicates specified in each figure or legend. The number of animals used was designed to maximise gaining physiologically relevant, statistically significant data, whilst minimising the numbers of animals used. We focussed on the key group of high fat fed female rats and data were gathered to reach statistical significance. This group of rats were more homogenous in response compared to control animals, and data reach statistical significance at n = 3 at between P < 0.001 and P < 0.0001 (see Figs. 1, 2, 3 and 4) using ANOVA with post hoc testing as described below.

While data from control rats is suggestive of response to cytokines in some cases (see Figs. 2 and 4) it did reach not reach statistical significance. It could be argued that greater numbers might detect significant change, but power calculations (https://www.stat.ubc.ca/~rollin/stats/ssize/n2.html) gave n = 16 rats needed to detect differences, which was deemed prohibitive for the aims of this study. Calculations were performed using GraphPad Prism 8 software (GraphPad Software, La Jolla, USA). A one-way ANOVA was used for comparison of initial control in vivo CSF secretion and resistance to CSF drainage experiments against treatment groups. A two-way ANOVA was used for comparison of in vivo control CSF secretion rates and resistance to drainage experiments for both diets, respectively. In all cases, ANOVAs were followed by an unpaired t test with Welch-correction (one-way ANOVA) or Sidak’s multiple comparison post hoc test (two-way ANOVA) to determine a significant difference among groups. The significant multiple comparison results following the post-hoc test is shown in each graph. Positive/negative results refer to an increase/decrease in CSF secretion rates over controls, respectively. Statistically significant differences are presented as probability levels of *P* < 0.05 (*), *P* < 0.01 (**), *P* < 0.001 (***), *P* < 0.0001 (****).

## Results

Idiopathic intracranial hypertension is a result of raised ICP, possibly due to increased CSF secretion, decreased drainage, or a combination of both.

### CSF secretion

It was important to test diet effects on CSF secretion rates in rats fed either C or HF diet due to the increasing incidence of IIH in all populations due to the rising obesity rates and several studies reporting weight gain in newly diagnosed IIH patients [22, 23].

Food and water intake are shown in Additional file 1: Fig. S1a and b, respectively. The average percentage weight gain over the 7-week period was significantly higher for the male HF diet rats (447.1%) when compared to the C diet (277.7%), as shown in Additional file 1: Fig. S2. A smaller but significant increase was observed when comparing the HF diet females (347.6%) with C diet female rats (265.7%) (Additional file 1: Fig. S3).

Cerebrospinal fluid secretion rates of HF diet females (3.18 ± 0.12 μl/min, n = 3) were significantly higher than in males fed either the C (1.57 ± 0.23 μl/min, *P *< 0.001, n = 3) or HF diet (2.06 ± 0.42 μl/min, *P* < 0.01, n = 4), as well as compared to females fed the C diet (1.49 ± 0.15 μl/min, n = 3, *P* < 0.0001) (Fig. 1). There was no difference between male and female rats when comparing CSF secretion rates in animals fed the C diet or between males on different diets, which suggests that the influence of HF diet on CSF secretion was more prominent in females (Fig. 1).

Rates of CSF secretion (shown as percentage of control) were increased in female rats fed both the C (131.7% ± 13.1%, n = 3) and HF (113.6% ± 1.3%) diet following HC treatment (Fig. 2). CSF secretion was also significantly increased in rats fed the C diet following TNF-α treatment (136.5% ± 5.0%, n = 3) (Fig. 2).

Treatment with IL-6, CCL2 and IL-17 did not alter rates of CSF secretion in female rats fed either C or HF diet (Fig. 2).

### Resistance to CSF drainage

The VRI method was used to test the in vivo effect of cytokine treatment on the resistance to CSF drainage over four increasing infusion rates (5, 10, 16, 20 μl/min). The resistance to CSF drainage was compared in untreated female animals on C and HF diets (Fig. 3). The results showed no significant differences between female rats fed a HF diet (7.1 ± 0.3 mmH_2_O min/μl, n = 3) and females fed the C diet (6.1 ± 0.4 mmH_2_O min/μl, n = 3). We therefore decided to test the effect of cytokines on resistance to CSF drainage on female rats raised on either a C or HF diet.

Treatment of rats fed a C diet with TNF-α and HC induced a decrease in resistance to CSF drainage compared to untreated rats (40.8% ± 39.7% and 62.5% ± 42.6%, n = 3 respectively), however this decrease did not reach statistical significance (Fig. 4).

There were no significant changes in resistance to CSF drainage in female rats fed either a C or HF diet following IL-6 or IL-17 treatment (Fig. 4). However, CCL2 induced a significant increase in females fed the C diet (140.6% ± 7.5%, n = 3, *P* < 0.001) and HF diet (138.9 ± 3.4%, n = 3, *P* < 0.0001) over the respective controls (Fig. 4).

## Discussion

In the current study, we provide an in vivo insight into potential mechanisms relating to the pathogenesis of IIH. We highlight increased CSF secretion rates in female rats fed a HF diet as well as following cytokine treatment in both diets (HC) and C diet (TNF-α). We also describe an increase in resistance to CSF drainage following CCL2 treatment in female rats on both diets, all of which provide a pathogenic link between weight gain and increased ICP in IIH.

### CSF secretion

The biggest increase in CSF secretion rates was seen in female rats fed on a HF diet regardless of treatment used.

The increased CSF secretion rates associated with HC may be consistent with the hypothesis that an increased activity of the 11β-HSD1 enzyme in CP epithelial cells leads to increased Na^+^ transport through the ENaC [19]. Indeed, HC increases the activity of the ENaC in retinal pigment epithelium of New Zealand White Albino rabbits [19] and it is possible that a similar mechanism operates in CP epithelium where three isoforms of ENaC have been detected [24]. In this putative pathway, corticosterone would be converted to cortisol through NADP(H) activation of the 11β-HSD1 enzyme in CP epithelium. The enzyme has been identified in CP and preferentially generates cortisol through oxo-reductase activity [19]. Sinclair et al. propose that cortisol would then bind to intracellular glucocorticoid receptors, activating serum glucocorticoid kinase 1 pathways to increase the movement of Na^+^ across the cells via ENaC route, creating an osmotic gradient in order to drive water into the CSF. The cellular location of ENaC and its involvement in Na^+^ and water movement across the CP epithelium has yet to be established however. For example, if the channel is on the basal (blood) face, it could facilitate Na^+^ movement into the epithelium, supporting Na^+^/K^+^ ATPase mediated Na^+^ flux to CSF, but it is difficult to see how that can be accomplished if ENaC is on the apical (CSF) face as suggested [19]. The 11β-HSD1 pathway, which may modulate CSF secretion rate, may also be induced by TNF-α. TNF-α up-regulates 11β-HSD1 enzyme through the secretion of phospholipase A2 in rat glomerular mesangial cells [25]. The joint perfusion of TNF-α and HC in the aCSF for example could be useful to investigate whether an additive effect of these treatments could be seen on CSF secretion rates.

Based on the results, the increased levels of cortisol in HF diet groups may mediate, at least partly, the increase in CSF secretion rates in rats fed a HF diet over rats raised on C diets within this study. Of the other inflammatory mediators tested, IL-6, IL-17 and CCL2, no changes were seen to CSF secretion in either control or HF rats. This does not of course, rule out other factors induced by a HF diet that may influence CSF dynamics in IIH patients with obesity. However, our results here suggest a collective treatment against HC elevation, possibly through inhibition of 11B-HSD1 activity, and a low-fat diet could be the main course of therapy for reducing raised ICP associated with female IIH patients with obesity following future studies.

In addition to obesity being a factor in the incidence of IIH, sex hormones may influence patients with IIH as it is found to occur mostly in premenopausal women with obesity [26], with a female to male ratio of 8:1 [1].

Endocrinological dysfunction within females of child-bearing age have been postulated as causes of increased ICP in female IIH patients [27]. Increased amounts of adipose tissue, also associated with obesity, acts as an endocrine organ, releasing hormones such as leptin, and produces increased levels of oestrogen via the conversion of androstenedione. This can lead to physiologically abnormal amounts of these hormones in a person’s body which may contribute to the development of IIH [27, 28] or symptoms of IIH [29]. There is evidence of increased oestrogen and prolactin hormone levels giving rise to cortisol and TNF-α, respectively, in females [30]. Female rats have a more intense corticosterone response to stress effect, (partially mediated by oestrogen) [31] which could offer a potential mechanism by which increased CSF secretion is more likely to be associated with female rats over males.

TNF-α is also elevated in healthy control subjects of human females over males [32]. Women generate high serum levels of anterior pituitary hormone prolactin, in response to stressful stimuli [30]. Prolactin is known to stimulate the immune system, enhancing proliferation and function of lymphocytes and macrophages which are cells that can secrete cytokines. Studies by Zhu have shown TNF-α plasma level increases following subcutaneous injection of prolactin in male mice [30]. This could be the reason why the CSF secretion rates on C diet female rats with TNF-α treatment were elevated in our study.

Treating elevated CSF secretion in IIH patients may be achieved through either serotonin to inhibit Na^+^–K^+^-ATPase pathway through the activation and phosphorylation of protein kinase C [33]; antisense thyroid transcription factor-1 oligodeoxynucleotide to reduce AQP1 mRNA and protein expression in the CP [34]; or acetazolamide and topiramate treatment [35] to decrease intracellular carbonic anhydrase CP epithelium [6]. However, reducing weight loss and inhibiting the actions of reproductive hormones as well as HC and may result in decreased CSF formation and ICP in IIH patients.

### Resistance to CSF drainage

A HF diet did not change resistance to CSF drainage. However, in terms of additional cytokine treatment CCL2 caused increased resistance in the both female groups. Overall, CCL2 in female rats fed a HF diet had the biggest impact on resistance to CSF drainage in vivo, possibly through this increased arachnoid resistance to CSF outflow. Obesity is an inflammatory condition where increased circulating or CSF cytokines may result in fibrotic changes or lead to a hypercoagulable state causing blockage of the arachnoid villi and, therefore reducing drainage of CSF [36]. This pathway is also often aggravated by thrombophilic exogenous oestrogens. In addition, further studies on the associations between CCL2 on inflammation of the arachnoid villi, hyperandrogenism and PCOS, may highlight a possible role in the cause of increased resistance to CSF drainage and elevated ICP in female IIH patients with obesity [37–39].

Decreases in resistance to CSF drainage were seen in the female rats fed a C diet following HC and TNF-α treatment (although not significant), which was not seen in the HF diet female group. Studies have shown an increase in the expression of AQP4 in the glymphatic pathway, due to the release of TNF-α, during parenchymal CSF absorption [40]. This may explain the tendency to decreasing resistance to CSF drainage associated with TNF-α in rats fed on a C diet. Whether this mechanism is altered in rats on a HF diet remains to be determined.

Overall, pro-inflammatory cytokines, especially CCL2, could potentially be used as diagnostic markers and may serve as targets for therapeutic intervention following further studies in larger cohorts. This being if they are found to alter CSF drainage pathways; cause elevated ICP through increased resistance to CSF drainage; and therefore contribute to IIH. Developing an inhibitory treatment against CCL2 elevation in patients with IIH could be advantageous in reducing this resistance of CSF drainage, possibly around the arachnoid granulations, and therefore lowering ICP.

## Conclusion

Weight loss and therapies targeting HC, TNF-α and CCL2, whether separately or in combination, may be beneficial to modulate rates of CSF secretion and/or resistance to CSF drainage pathways, both factors likely contributing to the raised ICP observed in female IIH patients with obesity.

## Supplementary information

**Additional file 1: Figure S1.** Food and water intake of male and female rats fed a C or HF diet. Graphs for food (**a**) and water (**b**) intake of male and female rats raised on either a C or HF diet are displayed. **Figure S2.** Average percentage weight gain of male rats fed a C or HF diet over a 7-week period. Readings were recorded weekly over a 7-week period (from 4 weeks of age) for both C and HF diet male rats. Average percentage weight gain was monitored prior to VCP and VRI experiments which were performed at 11 weeks of age. **Figure S3.** Average percentage weight gain of female rats fed a C or HF diet over a 7-week period. Readings were recorded weekly over a 7-week period (from 4 weeks of age) for HF diet rats and four week period (from 7 weeks of age) for C diet rats (extrapolated over literature female rat weights at week 0 (red line)). Average percentage weight gain was monitored prior to VCP and VRI experiments which were performed at 11 weeks of age.

## Data Availability

The datasets used and/or analysed during the current study are available from the corresponding author on reasonable request.

## Abbreviations

aCSF: artificial cerebrospinal fluid
AQP-1: aquaporin-1
CCL2: C–C motif chemokine ligand-2
CP: choroid plexus
CSF: cerebrospinal fluid
ENaC: epithelial sodium channel
HC: hydrocortisone
HF: high-fat diet
ICP: intracranial pressure
IIH: idiopathic intracranial hypertension
Na^+^–K^+^-ATPase: sodium potassium ATPase pump
NKCC1: Na^+^–K^+^–2Cl^−^ cotransporter
PCOS: polycystic ovarian syndrome
SAS: subarachnoid space
TNF-α: tumour necrosis factor-α
VCP: ventriculo-cisternal perfusion
VRI: variable rate infusion
