# Supplementary material for: Cerebrospinal fluid dynamics modulation by diet and cytokines in rats
Source: Fluids Barriers CNS. 2020 Feb 10;17:10. doi: 10.1186/s12987-020-0168-z (PMC7008525; doi:10.1186/s12987-020-0168-z)
Supplement: Supplementary file 1 — Additional file 1: Figure S1. Food and water intake of male and female rats fed a C or HF diet. Graphs for food (a) and water (b) intake of male and female rats raised on either a C or HF diet are displayed. Figure S2. Average percentage weight gain of male rats fed a C or HF diet over a 7-week period. Readings were recorded weekly over a 7-week period (from 4 weeks of age) for both C and HF diet male rats. Average percentage weight gain was monitored prior to VCP and VRI experiments which were performed at 11 weeks of age. Figure S3. Average percentage weight gain of female rats fed a C or HF diet over a 7-week period. Readings were recorded weekly over a 7-week period (from 4 weeks of age) for HF diet rats and four week period (from 7 weeks of age) for C diet rats (extrapolated over literature female rat weights at week 0 (red line)). Average percentage weight gain was monitored prior to VCP and VRI experiments which were performed at 11 weeks of age. [file 12987_2020_168_MOESM1_ESM.docx]

**Additional data**

**Food and water intake**

Food and water intake was monitored daily in order to determine the amount of nutrients the rats on each diet were obtaining, and whether food intake would affect the rat’s water intake. Food and water intake are shown in Fig. s1 (a) and (b), respectively.

**(b)**

**(a)**

Figure S1: Food and water intake of male and female rats fed a C or HF diet. Graphs for food (a) and water (b) intake of male and female rats raised on either a C or HF diet are displayed. C diet rats in both sexes were fed the ‘Rat and Mouse No. 1 Maintenance (RM1) control diet (n=3, respectively). HF diet male (n=4) and female (n=3) rats were fed the 45% AFE High Fat diet. Food and water intake were recorded as cage averages (±SD) as the rats were not housed singly. A one-way ANOVA was used to analyse the statistical significance. The significant results are shown following Sidak’s multiple comparison test against each diet variables. ** *P* <0.01. C: control diet; HF: high-fat diet *****P* <0.0001.

Food intake was significantly higher in the male rats raised on the C diet (20.43 ±1.24 g/day) compared to male rats on the HF diet (18.01 ±1.61 g/day, *P*<0.01) and female rats on both diets (C diet: 14.9 ±0.42 g/day, *P*<0.0001; HF diet: 13.7 ±0.63 g/day, *P*<0.0001) (Fig s1a). In addition, male rats raised on the HF diet also had s significantly higher food intake than the female rats on the C (*P*<0.001) and HF (*P*<0.0001) diet.

Female rats raised on the C diet had a significantly lower water intake than male rats raised on both diets (C diet 30.31 ±3.62 ml/day, P<0.01); HF diet 29.84 ±2.04 ml/day, P<0.001). There was no statistical significance between the female rats fed the HF diet (27.69 ±1.84 ml/day) and all other groups.

**Average percentage weight gain**

The average percentage weight gain between normal diet and HF diet was monitored to confirm that the HF diet induced weight gain in both male (Fig. s2) and female (Fig. s3) Wistar rats.

**Figure S2: Average percentage weight gain of male rats fed a C or HF diet over a 7-week period.** Readings were recorded weekly over a 7-week period (from 4 weeks of age) for both C and HF diet male rats. Average percentage weight gain was monitored prior to VCP and VRI experiments which were performed at 11 weeks of age. C diet (dashed line), HF diet (solid line). An unpaired t test was used to measure statistical significance between the two groups at each week. *****P* = <0.0001. C: control diet; HF: high-fat diet; VCP: ventriculo-cisternal perfusion; VRI: variable rate infusion.

**Figure S3:** **Average percentage weight gain of female rats fed a C or HF diet over a 7-week period.** Readings were recorded weekly over a 7-week period (from 4 weeks of age) for HF diet rats and four week period (from 7 weeks of age) for C diet rats (extrapolated over literature female rat weights at week 0 (red line)). Average percentage weight gain was monitored prior to VCP and VRI experiments which were performed at 11 weeks of age. C diet (dashed line), HF diet (solid line). An unpaired t test was used to measure statistical significance between the two groups at each week. *****P* = <0.0001. C: control diet; HF: high-fat diet; VCP: ventriculo-cisternal perfusion; VRI: variable rate infusion.

Initially, C diet fed male rats had a higher percentage weight gain after week 1 over HF fed male rats (*P* < 0.0001). However, the average percentage weight gain was statistically higher over the duration of the 7-week period for the male rats raised on the HF diet (447.14%) when compared to the rats on the C diet (277.68%) which started increasing by week 2 (*P* <0.0001) (Fig. s2). The same overall correlation was seen, although at lower levels, with the female rats fed the HF diet (347.57%) when compared with females raised on the C diet (265.65%) (*P* <0.0001) (Fig. s3).
